# Determining the Glycaemic Index of Standard and High-Sugar Rodent Diets in C57BL/6 Mice

**DOI:** 10.3390/nu10070856

**Published:** 2018-07-01

**Authors:** Grace J. Campbell, Damien P. Belobrajdic, Kim S. Bell-Anderson

**Affiliations:** 1 Charles Perkins Centre, School of Life and Environmental Sciences, University of Sydney, Sydney, NSW 2006, Australia; kim.bell-anderson@sydney.edu.au; 2 Commonwealth Scientific and Industrial Research Organisation (CSIRO), Health and Biosecurity, Adelaide, SA 5000, Australia; damien.belobrajdic@csiro.au

**Keywords:** glycaemic index, mice, rodent diets, sugar, carbohydrate

## Abstract

The glycaemic index (GI) is a useful tool to compare the glycaemic responses of foods. Numerous studies report the favorable effects of low GI diets on long term metabolic health compared with high GI diets. However, it has not been possible to link these effects to the GI itself because of other components such as macronutrients and dietary fibre, which are known to affect GI. This study aimed to create and evaluate isocaloric diets differing in GI independent of macronutrient and fibre content. The GIs of eight diets differing in carbohydrate source were evaluated in mice; cooked cornstarch (CC), raw cornstarch (RC), chow, maltodextrin, glucose, sucrose, isomaltulose, and fructose. A glucose control was also tested. The GIs of all eight diets were different from the GI of the glucose control (GI: 100; *p* < 0.0001). The GIs of the glucose (mean ± SEM: 52 ± 3), maltodextrin (52 ± 6), CC (50 ± 4), RC (50 ± 6), and chow (44 ± 4) diets were similar, while the GIs of the sucrose (31 ± 4), isomaltulose (24 ± 5), and fructose (18 ± 2) diets were lower than all other diets (*p* < 0.05). This is the first trial to report GI testing in vivo in mice, resulting in three main findings: chow is relatively high GI, the glucose availability of raw and cooked cornstarch is similar, and the GI of different sugar diets occur in the same rank order as in humans.

## 1. Introduction

Obesity is on the rise worldwide, reaching epidemic proportions [1,2]. One of the major factors in developing obesity is poor dietary choices. The industrialization of food processing has changed the available foods in western society, leading to an increase in ultra-processed foods, such as refined starchy foods, and a subsequent increase in energy from glucose [3]. One way of characterizing the carbohydrate quality of foods is by the glycaemic index (GI). The GI is a measure of the rate of change in blood glucose levels following a meal [4,5]. Low GI meals maintain a stable blood glucose level, without a dramatic peak, and are associated with improved weight management, insulin sensitivity, reduced HbA_1C_, and lowered risk of type 2 diabetes mellitus when followed for a minimum of six months (reviewed in [6]), but the mechanism(s) responsible have not been fully elucidated.

Typically, high GI foods result in larger postprandial blood glucose excursions compared with low GI foods, and this is assumed to be responsible for the associated negative health outcomes through extended hyperinsulinaemia and hyperglycaemia causing long-term multi-organ stress. Thus, low GI diets are recommended as a management strategy for people with type 2 diabetes [7,8,9,10,11]. However, the differences between high and low GI diets are generally quite complex, for instance, low GI diets are associated with high fibre content, which produces distinctly separate health benefits [12]. Additionally, high fat and high protein diets can be low GI because of the decrease in carbohydrate content, but these diets may not be metabolically favorable. Low GI diets can also contain sugars such as fructose. Fructose is a low GI sugar that contributes approximately 9% of the total energy consumed by the average American [13]. Yet, despite being low GI, there exists controversy surrounding the effect of fructose and other sugars on metabolic health [14]. Untangling these complex dietary interactions in order to isolate the mechanism(s) of GI itself is difficult in human studies because of the necessary nutrient requirements and huge cost of providing controlled meals over an extended period of time. For this reason, animal studies can be employed, where the exact composition of the diet can be completely controlled. Yet, there are still confounding issues.

In many rodent studies evaluating the effects of high GI diets versus low GI diets, the low GI diets contain high levels of resistant starch. The exact breakdown of this resistant starch was rarely given, but all contained a significant portion that would be fermented in the large intestine as fibre. Subsequently, the low GI diets contain much higher levels of dietary fibre, making it difficult to determine whether the metabolic changes are because of the lower glycaemic impact of the diet, the dietary fibre content, or both. In a recent meta-analysis, we concluded that it was not yet possible to elucidate the molecular mechanisms of high GI diets in rodents because of the heterogeneity of methodologies, most notably differences in other dietary components [15]. Only one of the thirty papers examined controlled for both macronutrient and fibre content, but these rats were only studied for two weeks, producing limited metabolic results [16]. Some studies attempted to test the GIs of their diets, but no study did so in a methodologically rigorous way.

Therefore, the aim of this study was to create and evaluate isocaloric, high-carbohydrate diets matched for both macronutrient and fibre composition that differed only in GI. To do this, we established a GI testing protocol in mice that was adapted from the clinical standard [17]. The test diets were designed to be identical, only differing in the type of carbohydrate. Four diets were made using different simple sugars that have been shown to cover a wide GI range when fed to humans as a pure sugar; glucose (GI: 100), sucrose (65), isomaltulose (32), and fructose (19) [18,19]. The simplicity of these sugars facilitates complete digestion in the small intestine, reducing confounding effects of microbiota due to the fermentation of fibre. The glucose polysaccharide maltodextrin was added to all diets to slightly raise the overall glycaemic response. This was deemed necessary as otherwise the fructose diet would contain no glucose, and thus the blood glucose levels may not noticeably rise. In addition, a maltodextrin diet was also included to confirm that maltodextrin is high GI when digested in mice, and thus will raise the GI of the diets as designed. An additional two starch-based diets were tested (raw and cooked cornstarch) as these carbohydrates are frequently used as control rodent diets, as was the commonly used standard chow diet.

## 2. Materials and Methods

The GIs of eight diets were tested in vivo, in a cross-over design, in ten C57BL/6 female mice following the standard for GI testing in humans [17]. Ten six-week-old female C57BL/6 mice were purchased from Australian BioResources (ABR, Moss Vale, NSW, Australia). The mice were housed five per cage in reverse light cycle conditions, lights on 5:45 p.m. to 5:45 a.m., at 21 °C and 60% humidity in the animal house at the Charles Perkins Centre, University of Sydney. The mice were meal trained over approximately 16 weeks and were provided 0.3 g of available carbohydrate of the test diet during the tests. Eight diets and one glucose control were tested: standard chow, raw (RC), and cooked cornstarch (CC), and diets high in the following sugars: maltodextrin, glucose, sucrose, isomaltulose, and fructose.

All animal procedures were approved by the University of Sydney Animal Ethics Committee protocol number 2015/814.

### 2.1. Meal Training

The mice were acclimatized to the facility for one week on *ad libitum* standard chow diet. Following acclimatization, mice were fed at 7 a.m. and 4 p.m. every day. Initially, they were exposed to each of the test diets for one day, 7 a.m.–4 p.m., with chow provided at 4 p.m. until the following day. To start the training, mice were placed in individual cages for both meals and allowed to eat chow for two hours. It quickly became apparent that mice were not eating sufficiently, so the 4 p.m. feedings were changed to be group feedings of five mice per cage, to induce competition between the mice. Hereafter, the 7 a.m. (beginning of dark phase) meal will be referred to as the ‘individual’ feeding time, and the 4 p.m. (end of dark phase) meal will be referred to as the ‘group’ feeding time. After six days, the meal time was halved and mice were allowed to eat for 1 h at both time points. An additional six days later, the time food was available was reduced to 30 min. It was determined that 1 h total of food access was the minimum amount of time to ensure sufficient feed intake. After another six days, the individual meal time was dropped for a final time to 15 min, and correspondingly, the group meal time was increased to 45 min. These meal lengths were maintained for the duration of the study. The mice were weighed thrice weekly from diet exposure, and daily once meal training began.

### 2.2. GI Testing

GI testing was performed once the mice were trained to reliably eat individually within 15 min at the start of the dark phase. GI testing was conducted every six days, with animals being fed chow for the five intervening days. On test days, mice were placed individually in a clean cage without bedding, but containing environmental enrichment. To obtain a drop of blood, the end of the mice’s tails were cut with a scalpel blade. The blood droplet was tested using an Accu-Chek Performa glucometer (Roche Diabetes Care, Basel, Switzerland). The mice were allowed to settle for 15 min and then blood glucose levels were measured. If the lowest blood glucose reading was below 8.5 mM and the animal showed no visual distress signs, this blood glucose level was considered the basal level and the test began. The mouse was randomly provided a portion of the glucose control or a test diet on a petri-dish, with the serve size calculated to provide 0.3 g of carbohydrate. The petri-dish and any remaining food was removed after 12 min. Blood glucose levels were again checked with a glucometer at 15, 30, 45, 60, 90, and 120 min after provision of the food. If all food was consumed, the results were calculated. Otherwise, the test for that mouse with that test food was repeated after all diets had been tested at least once. Glucose control testing had to be repeated considerably more than most, despite being the smallest meal by weight, most likely because of mouse preference. Test repeats were randomized to a point, allowing for different mice requiring different tests and minimizing the duplicate tests on the same day. The usual chow was provided at 4 p.m. on the day of testing.

### 2.3. Meal Composition

The glucose control was purchased as Glucodin Energy Tablets (Chemist Warehouse, Virginia, QLD, Australia), and chow was provided from the animal house purchased from Specialty Feeds (‘meat-free rat and mouse diet’, Glen Forest, WA, Australia) containing 65% carbohydrate, 23% protein, and 12% fat by energy content and 59.4% carbohydrate by weight. The other seven diets were made in-house as described in Table 1, based on the standard American Institute of Nutrition AIN-93 formulation, and were all approximately 64% carbohydrate, 22% protein, and 14% fat by energy content [20]. All of the diets, excluding the CC diet, were prepared by mixing all ingredients and then adding water to 5 g of the diet until it could be compressed into a ball. The diets were dried down in a fume-hood overnight, and serve size calculated such that it contained 0.3 g of carbohydrate. The CC was prepared by slowly adding cornstarch to boiling water in a glass beaker on a hot plate such that it gelatinized, ensuring the water resumed boiling between each addition of cornstarch. It was then left to cool for 20 min before being mixed, by kneading, with the remaining diet ingredients before drying in a fume-hood overnight in the same way as the other diets.

### 2.4. Statistics

The GIs were calculated for each diet in each mouse by comparison of the incremental blood glucose area under the curve of the test diet and the glucose control, using the incremental area under the curve (iAUC) method, and averaged across the 10 mice [21]. All graphs were created in the statistical programming language R using the gglpot2 and dplyr packages [22,23,24]. The GIs and 15 min peak blood glucose values were analyzed by analysis of variance (ANOVA) and Student’s *t*-test in R, with statistical significance at *p* < 0.05. Values are expressed as mean ± standard error of the mean.

## 3. Results

### 3.1. Body Weight

**Figure 1:**
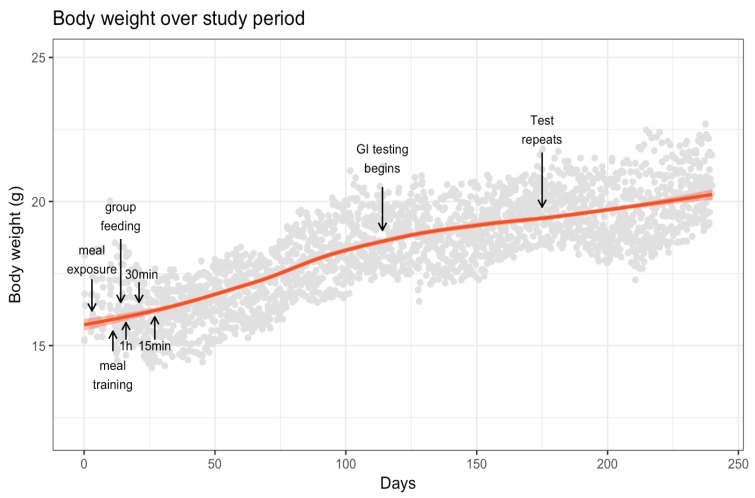
Smoothed mean mouse body weight across the study. Arrows and annotations in panel indicate changes in feeding: day 3, started meal exposure; day 11, started meal training; day 14, started group feeding for the 4 p.m. meal; day 16, meal time decreased to 1 h; day 21, meal time reduced to 30 min; day 27, meal time reduced to 15 min; day 114, started actual tests; day 175, test repeats started; day 244, last test. GI—glycaemic index.

The mouse body weights for the duration of the study are shown in Figure 1. The weights for all 10 mice decreased upon starting meal training, but slowly began to rise within 10 days as the training pattern was established. Body weight varied throughout the study as a result of individual mouse behavior, but all mice trended to increase their body weight over time as expected. A mouse was to be removed from training and given chow *ad libitum* if it lost more than 15% chronically or 10% acutely of its bodyweight, based on the Guidelines to Promote the Wellbeing of Animals Used for Scientific Purposes (Nation Health and Medical Research Council, Canberra, ACT, Australia), but no mouse reached this limit.

### 3.2. Food Intake

**Figure 2:**
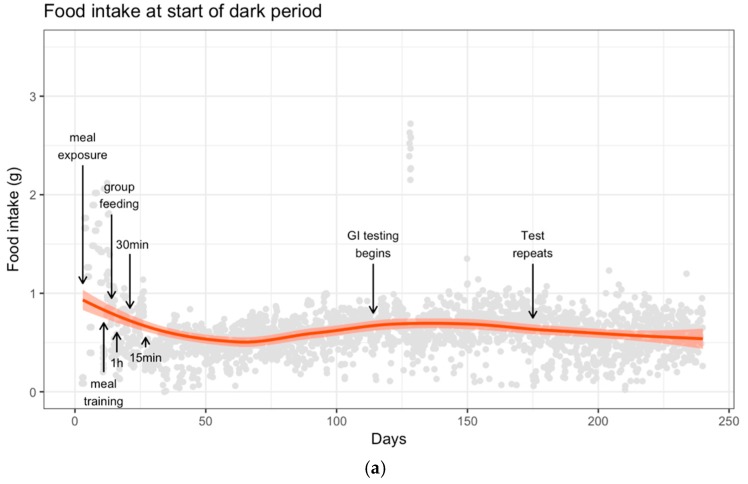

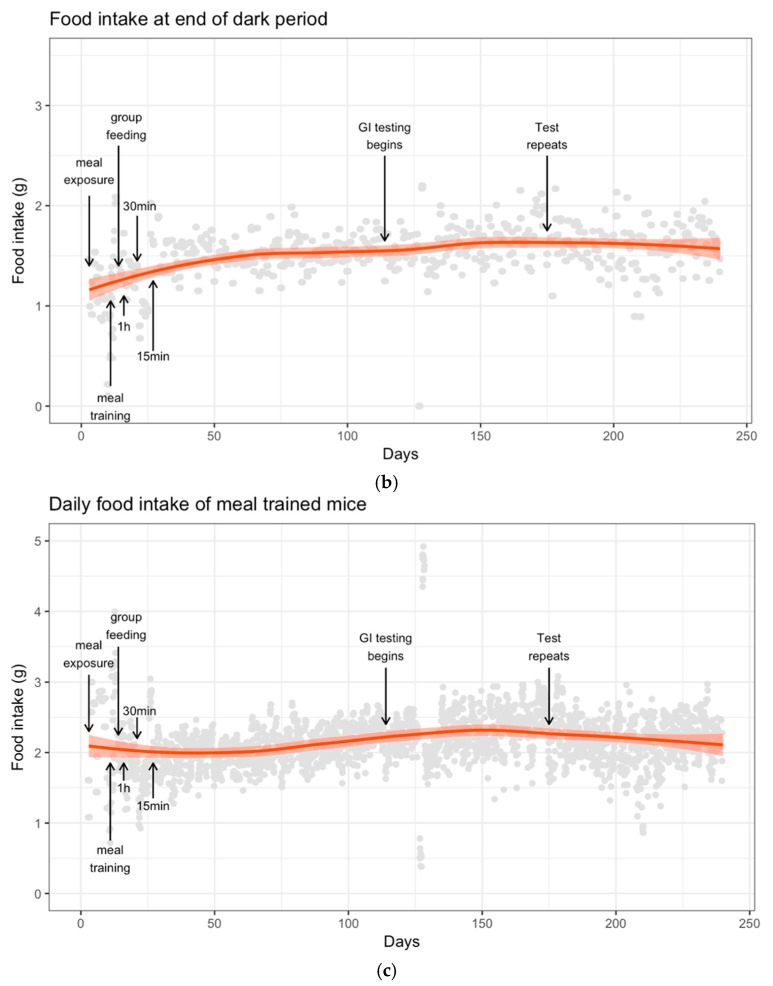
Smoothed mean of food intake (**a**) beginning of dark phase (7 a.m.—individual); (**b**) end of dark phase (4 p.m.—group); and (**c**) daily for the ten mice across the 250-day study. Mice were group fed for the vast majority of 4 p.m. feedings, but individually during the 7 a.m. meals (grey dots).

The food intake for both individual and group meals was recorded for each mouse (Figure 2). After food access was restricted, there was a considerable reduction in intake from 2.3 ± 0.2 g to 1.9 ± 0.1 g per mouse per day. However, once a steady schedule was reached, the mice maintained their intake, allowing for fluctuations due to animal behavior.

At approximately day 125, there was one period of reduced food intake due to an error in the feeding protocol, resulting in a small and transient drop in body weight. Subsequently, the mice were provided with food for 2 h the following morning, which restored most of the lost weight. The meal training protocol was adjusted for the next few days to allow for the mice to recover and they were back to the normal schedule and weight within a week.

### 3.3. GI Tests

**Figure 3:**
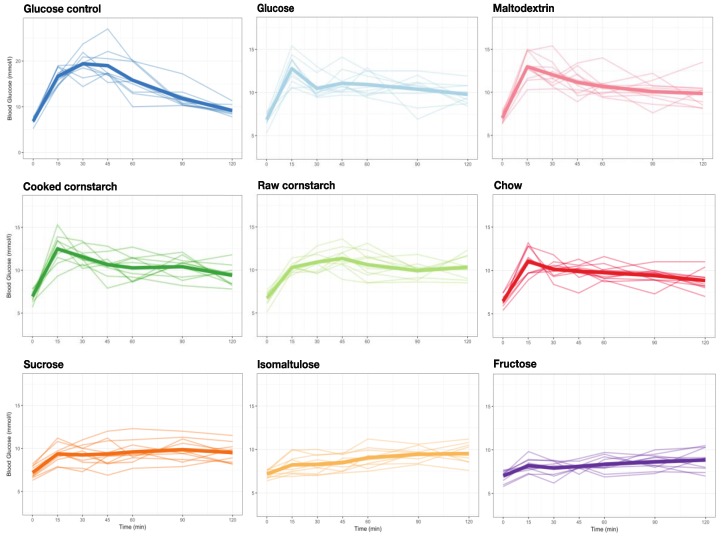
Line graphs of individual mouse blood glucose response to eight diets (containing 0.3 g carbohydrate) and glucose control (0.31 g) over two hours. *n* = 10 female mice for each diet.

**Figure 4:**
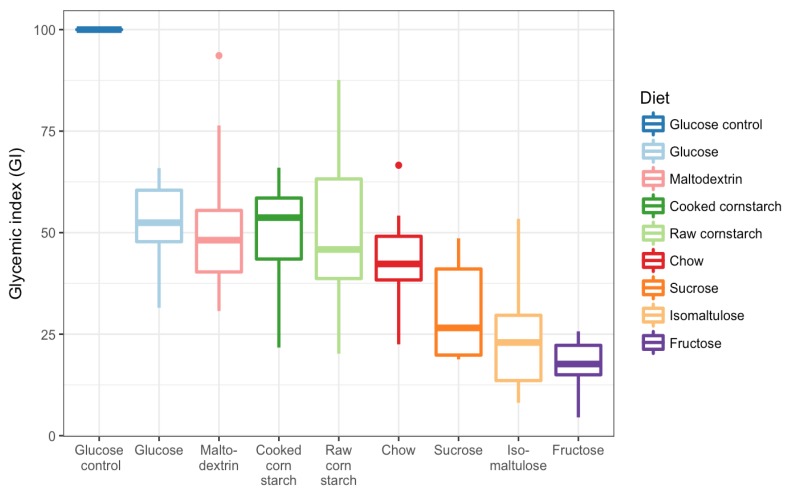
Boxplot of glycaemic index of eight diets (all ~60% carbohydrate as energy) differing in type of carbohydrate and glucose control. *n* = 10 female mice for each diet.

Each of the ten mice successfully completed all nine tests. The glucose control GI test was repeated on average four times, and the CC and chow diet tests were repeated an average of once, per mouse. Fructose and RC diet tests were repeated three times overall; the isomaltulose diet test only once; and the glucose, maltodextrin, and sucrose diets were not repeated. The blood glucose curves for each mouse, for each diet, and the glucose control are shown in Figure 3. From these curves, the GI of each of the diets could be calculated (Figure 4). The GI of all eight meals were significantly different from the glucose control (GI: 100; *p* < 0.0001). Glucose (52 ± 3), maltodextrin (52 ± 6), CC (50 ± 4), RC (50 ± 6), and chow (44 ± 4) diets were not significantly different. Sucrose (31 ± 4), isomaltulose (24 ± 5), and fructose (18 ± 2) diets were also not significantly different, but were significantly different from the other diets (glucose, maltodextrin, CC, *p* < 0.05, versus sucrose; glucose, maltodextrin, *p* < 0.001, CC, RC, *p* < 0.001, chow, *p* < 0.05, versus isomaltulose; glucose, maltodextrin, CC, RC, *p* < 0.0001, chow, *p* < 0.01, versus fructose).

**Figure 5:**
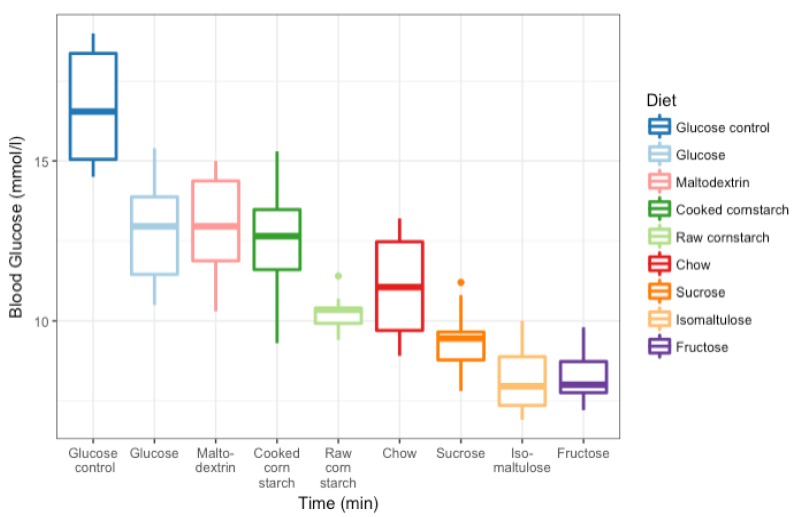
Boxplot of 15 min peak blood glucose during GI testing of eight diets differing in type of carbohydrate and glucose control. *n* = 10 female mice for each diet.

Despite similarities in GI, there were distinct differences in 15 min peak blood glucose levels during GI testing (Figure 5). The glucose control (average 15 min peak of 16.7 ± 0.6 mM) was significantly different from all diets (*p* < 0.0001). Maltodextrin (13.0 ± 0.5 mM), glucose (12.8 ± 0.5 mM), CC (12.5 ± 0.5 mM), and chow (11.0 ± 0.5 mM) diets were not significantly different from each other. RC (10.3 ± 0.2 mM) was significantly different from maltodextrin, glucose, and CC diets, but not chow (maltodextrin, glucose, *p* < 0.01; CC, *p* < 0.05). Sucrose (9.4 ± 0.3 mM), isomaltulose (8.2 ± 0.4 mM), and fructose (8.2 ± 0.3 mM) diets were not significantly different from each other, but were significantly different from the other diets (maltodextrin, glucose, CC, *p* < 0.0001, versus sucrose; maltodextrin, glucose, CC, *p* < 0.0001, versus isomaltulose; chow, *p* < 0.001, versus isomaltulose; maltodextrin, glucose, CC, *p* < 0.0001, versus fructose; chow, *p* < 0.001, versus fructose; RC, *p* < 0.05, versus fructose). The most notable result from this is the difference between RC and CC despite no difference in GI, suggesting that the single number of GI may not be sufficient to categorize the digestibility of a diet.

## 4. Discussion

Here, we report for the first time the in vivo GI values for rodent diets varying in simple sugar type and starch content, independent of their fibre and macronutrient content. We were successfully able to create and characterize isocaloric diets differing only in GI that can be utilized in future studies to potentially isolate the mechanism of GI. The order of the GIs of the high-sugars diets were consistent with the GIs reported for the base sugars tested in humans [18,25,26,27]. From the GIs of the diets, the glycaemic load could also be calculated, as the product of the GI and the carbohydrate content [28]. However, because of the similar carbohydrate content, the glycaemic loads for these diets would simply be a linear transformation of the GIs and would not provide any additional result. The glucose control was significantly higher than any of the meals, as expected, as it did not contain other nutrients such as protein, fat, and fibre, which are known to lower the rate of glucose digestibility and absorption [29]. The GI value for the chow diet was relatively high GI, and similar to the semi-purified diet that contained 60% glucose, meaning that it may not be as healthy a control as currently perceived. Similarly, the cornstarch diets had a high GI value. Interestingly, there was no difference between the calculated GI of the RC and CC diets, suggesting that cooking the cornstarch appeared to have no effect on the GI, despite gelatinization previously being shown to raise the GI and the initial rate of glucose absorption of each diet being significantly different [30]. During preparation of the CC diets, the temperature of the cooked cornstarch and boiling water mixture was checked with a thermometer at the start, three points during the addition of the cornstarch, and after all the cornstarch was added to make sure it was hydrolyzing, with readings occurring between 91 and 97 °C. As the mixture was always above 90 °C, and the cornstarch lost its white color, turning into a clear opaque gel-like paste, it can be assumed that the cornstarch did indeed gelatinize [31,32]. However, cooking and cooling temperature and time may have an effect on starch retrogradation, and thus different cooking techniques could have resulted in different GIs. Additionally, because of the different enzymes utilized for digestion, it is important to note that isomaltulose being completely digested in the small intestine has been confirmed in rats and pigs [33]. Thus, the differences in GI between isomaltulose and sucrose diets must be because of the different bonds between the fructose and glucose molecules, rather than remaining undigested isomaltulose.

We were successfully able to create and maintain mice on a meal training protocol in order to GI test rodent diets. An important consideration was to ensure that mice consumed sufficient quantities of food to sustain body weight. It was found that a 15 min individual session at the start of the dark phase was required to ensure each mouse wholly consumed the test food within 12 min during the test, although as a result of palatability, some test diets did necessitate repeating. We thus had flexibility in our group feeding at the end of the dark phase. For the first three days, mice were fed individually at both time points, but mice began to lose weight rapidly, so to combat this, the 4 p.m. feeds were changed to be consumed in their home cages of five mice. The competition this created led to a significant food intake increase and was continued for the rest of the study. We also found that 45 min in the group feedings paired with a 15 min individual session was optimal for our protocol. The two main considerations were that mice had enough time to eat to put on weight and be healthy, but time was minimized to ensure the mice were hungry enough to eat in the following individual meal session, rather than just wait until their group feed. An alternative meal test protocol in rats was recently developed that observed the physiological effects of carbohydrates that differed significantly to ours [34]. This protocol involved taking blood samples during presentation of different amounts of carbohydrate and different stages in the light cycle to measure insulin and glucose. It contrasts to our protocol in the level of invasion required of the rat, as theirs required anaesthetizing the rat to insert cannula for returning the rats’ red blood cells, as a large blood volume was taken, which would not be possible in mice. The short duration and lack of strict food restriction during the meal training protocol would also have resulted in issues if one of the test foods was less palatable to the rats. Interestingly, the blood glucose response was significantly decreased in the rat study compared with our mice, despite using four times the amount of available carbohydrate in some of the rat tests. This difference may be due to the differences in mice and rat weight as rats are typically at least ten times the size of a mouse; but weight data is not shown in the rat study, so this cannot be confirmed as the cause. However, the rat study showed that performing the GI testing at the start of the active (dark) phase was preferable, as is conducted in humans and in our study, and used similar amounts of carbohydrate for their smaller meal tests, as was used in our study.

A limitation of this study is that all mice used were female. Although there is limited evidence in animal models describing the effect of glucose control in male and females, clinical evidence suggests there are distinct metabolic differences between the sexes [35,36]. Given that a majority of published dietary intervention studies have been conducted on male rodents, it would be worth conducting follow up studies with both male and female mice. Similarly, in order to strengthen the predictability and utility of this GI testing model, it is important to conduct further testing in different strains and ages of mice, and perhaps with rats as well, though the amounts given to rats would be significantly larger. It may also be interesting to measure insulin and other hormones during GI testing, as is done in humans, although this would require larger blood sampling, and thus a more invasive protocol.

## 5. Conclusions

A mouse protocol emulating the GI testing procedure utilized in humans was successfully created and used to test isocaloric diets differing only in GI, independent of fibre content, which can be used for future long-term studies evaluating the metabolic effects of GI without confounding factors such as fibre. We also showed that chow may not be a healthy control in relation to GI, and that GI alone may not be sufficient to categorize all carbohydrates, for instance the similarities between the raw and cooked cornstarch diets GIs do not account for the significantly different blood glucose peaks or possible starch retrogradation. This GI protocol also has significant potential future uses, such as the GI testing of foods that cannot be achieved in humans, including when there is a limited availability of a novel food or food containing ingredients not approved for human consumption.

## Figures and Tables

**Table 1:** Diet components, based on a modified AIN-93 diet [20]. AIN—American Institute of Nutrition; RC—raw cornstarch; CC—cooked cornstarch.

INGREDIENTS (G/KG)	RC	CC	MALTODEXTRIN	GLUCOSE	SUCROSE	ISOMALTULOSE	FRUCTOSE
**CORNSTARCH**	600	600	-	-	-	-	-
**MALTODEXTRIN**	-	-	600	120	120	120	120
**GLUCOSE**	-	-	-	480	-	-	-
**SUCROSE**	-	-	-	-	480	-	-
**ISOMALTULOSE**	-	-	-	-	-	480	-
**FRUCTOSE**	-	-	-	-	-	-	480
**CALCIUM CASEINATE**	200	200	200	200	200	200	200
**SAFFLOWER OIL**	70	70	70	70	70	70	70
**WHEAT BRAN**	50	50	50	50	50	50	50
**AIN-93M MINERAL MIX**	45	45	45	45	45	45	45
**GELATINE**	15	15	15	15	15	15	15
**AIN-93 VITAMIN MIX**	13	13	13	13	13	13	13
**CHOLINE BITARTRATE**	4	4	4	4	4	4	4
**DL-METHIONINE**	3	3	3	3	3	3	3
**CARBOHYDRATE (%) ^A^**	55	55	59.2	62.1	62.1	59.5	62.1
**WATER: DIET (G)**	7:10	9:5	3:20	1:5	3:20	1:5	1:10

^A^ Calculated based on the carbohydrate content of all ingredients.

## Abbreviations

AIN	American Institute of Nutrition
CC	cooked cornstarch
GI	glycaemic index
iAUC	incremental area under the curve
RC	raw cornstarch
